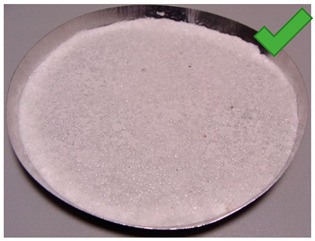# The Effect of Adhesive Additives on Silica Gel Water Sorption Properties

**DOI:** 10.3390/e22030327

**Published:** 2020-03-12

**Authors:** Karol Sztekler, Wojciech Kalawa, Agata Mlonka-Medrala, Wojciech Nowak, Łukasz Mika, Jaroslaw Krzywanski, Karolina Grabowska, Marcin Sosnowski, Marcin Debniak

**Affiliations:** 1 Faculty of Energy and Fuels, AGH University of Science and Technology, A. Mickiewicza Av.30, 30-059 Krakow, Poland; kalawa@agh.edu.pl (W.K.); amlonka@agh.edu.pl (A.M.-M.); wnowak@agh.edu.pl (W.N.); lmika@agh.edu.pl (Ł.M.); 2 Faculty of Science and Technology, Jan Dlugosz University in Czestochowa, Armii Krajowej 13/15, 42-200 Czestochowa, Poland; j.krzywanski@ujd.edu.pl (J.K.); k.grabowska@ujd.edu.pl (K.G.); m.sosnowski@ujd.edu.pl (M.S.); 3 Innogy Polska S.A. ul. Wlodarzewska 68 02-384 Warszawa, Poland

**Keywords:** adsorption chiller, coated beds, silica gel, COP, adsorption bed

## Abstract

Adsorption chillers are characterized by low electricity consumption, lack of moving parts, and high reliability. The disadvantage of these chillers is their large weight due to low adsorbent sorption capacity. Therefore, the attention is turned to finding a sorbent with a high water sorption capacity and enhanced thermal conductivity to increase chiller efficiency. The article discusses the impact of selected adhesives used for the production of an adsorption bed in order to improve heat exchange on its surface. Experiments with silica gel with three commercial types of glue on metal plates representing heat exchanger were performed. The structure of samples was observed under a microscope to determine the coverage of adsorbent by glue. To determine the kinetics of the free adsorption, the amounts of moisture adsorbed and the desorption dynamics the prepared samples of coated bed on metal plates were moisturized and dried in a moisture analyzer. Samples made of silica gel mixed with the adhesive 2-hydroxyethyl cellulose, show high adsorption capacity, low dynamic adsorption, and medium dynamic desorption. Samples containing adhesive poly(vinyl alcohol) adsorb less moisture, but free adsorption and desorption were more dynamic. Samples containing the adhesive hydroxyethyl cellulose show lower moisture capacity, relatively dynamic adsorption, and lower dynamic desorption.

## 1. Introduction

The demand for air conditioning units in the world in 2017 has increased to over 110 million units, which is an increase of 8.1% compared to the previous year. The most significant increase is observed in China (13.2%), Japan (6.5%), and Europe (8.1%) [1]. The sudden increase in demand for air conditioners or electric fans for households caused an increase in electricity consumption for these purposes, which currently accounts for about 20% of the global electricity consumption in the household sector; moreover, this share is continuously increasing, especially in countries with warmer climates. The decreasing prices of cooling units and increasing standard of living, the numbers of these units are continually increasing, which increases the comfort of life and work efficiency but has a significant impact on the energy demand of many countries, increases energy peaks, and significantly affects local and global pollution [2]. The growing demand for energy for cooling not only increases the load on the systems but also the amount of emitted pollutants. CO_2_ emissions during electricity production for refrigeration have tripled since 1990 to 1130 million tons in 2016, which can be compared with Japan’s total emissions. Besides, there is a high risk of environmental pollution with refrigerants, which can be toxic or have a greater impact on climate change than carbon dioxide. Most cooling systems consume electricity, especially compressors, pumps, and fans that consume a lot of electricity. Depending on the climatic conditions, the global instantaneous electricity consumption for ventilation, air conditioning, and refrigeration purposes may range from 30% to 50% of the total electricity consumption [1]. According to researchers, the electricity consumption for refrigeration in the European Union reached 8% in 2008 and is rapidly increasing. Most refrigeration systems are based on compressor refrigeration systems; adsorption chillers have significantly lower electricity consumption than compressor systems [3].

### 1.1. Adsorption Chillers

Adsorption chillers use heat from external sources to provide a cooling effect. Their main feature is the use of the working medium and the adsorber, which form the adsorption unit. The adsorption unit operating in an appropriate working system forms a thermal compressor. As opposed to an ordinary compressor, these are thermally driven, which means that heat rather than mechanical energy is supplied. The main advantage of such compressors is the fact that they can be driven with heat (often low-temperature heat). These chillers can be divided into absorption chillers, where the refrigerant is absorbed in the absorber, and adsorption chillers, where the refrigerant is absorbed on the surface.

**Figure 1:**
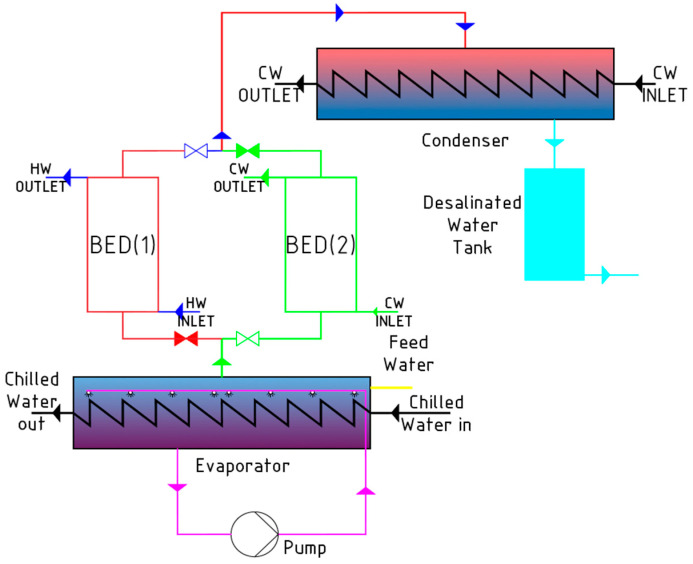
The principle of operation of adsorption chillers, according to [4].

The operation of the adsorption chiller system (Figure 1) involves evaporating the refrigerant in the evaporator at reduced pressure. The process takes place without the use of a mechanical compressor and uses the thermal effect of adsorption and desorption. The system operates cyclically; the adsorption and desorption (bed regeneration) processes take place alternately.

The refrigerant vapors discharged from the evaporator are adsorbed in the adsorption bed, which is accompanied by heat generation. Then, the absorption heat is picked up by the cooling water. To enable further bed operation, it is necessary to remove the refrigerant (adsorbate) from the adsorbent (desorption). This process requires heat supply from an external source. The refrigerant vapors released during the desorption process are discharged to the condenser. In a closed system, the condensate from the condenser goes back to the evaporator. The critical parameter determining the possibility of combining the adsorption chiller with the heat source is the bed regeneration temperature. The low desorption temperature allows for the more widespread use and utilization of low-temperature heat. The operation of the adsorption chiller with silica gel–water working pair is possible from about 43 °C in the case of two-stage devices and 62 °C in the case of single-stage devices [5].

The most commonly used adsorbent–adsorbate pairs include: zeolites–water, silica gel–water, activated carbon–ammonia, activated carbon–methanol, and calcium chloride–ammonia [6,7]. Due to the low desorption temperature, low costs, or lack of negative impact on the environment, devices based on adsorption technology often use silica gel–water pair. Silica gel has a fairly large specific surface (wide-porous: 250–350 m^2^/g, narrow-porous: 600–850 m^2^/g), allowing adsorbing large amounts of refrigerant vapor [8].

Addiotional benefitial aspect of adsorption technology is possibility of sea water desalination, what is a subject of a grate interest nowadays. Most recent studies analyse hybrid systems to enhance the recovery and decrease the overall desalination cost. A tri-hybrid system consisting of reverse osmosis pre-treatment facility and multi-evaporators integrated with adsorption cycle was proposed in [9] to enhance overall recovery up to 81%. Another hybrid system, multi-effect distillation (MED) and adsorption desalination (AD) hybrid desalination process was investigated in [10].

### 1.2. Methods to Maximize the Cooling Efficiency of the Adsorption Chiller

Research is currently underway to increase the refrigeration efficiency (COP) of adsorption chillers. The objective is to reduce the size of the device while maintaining the same cooling capacity. The actions aimed at maximizing cooling efficiency are as follows.

#### 1.2.1. The Adjustment of Adsorption and Desorption Time

Numerous studies are aimed at optimum operating conditions of two- and three-bed adsorption chillers. The desorption process is up to 2–3 times shorter than adsorption; therefore, some studies indicate that effective desorption time can be up to seven times shorter at high temperatures [11].

#### 1.2.2. Increasing the Number of Beds

One of the most common methods of improving the efficiency of an adsorption aggregate is to increase the number of adsorption beds to achieve the shortest intermittent cold production.

#### 1.2.3. The Intensification of Heat Exchange in the Bed

Increasing the thermal conductivity in the bed, especially in the layer between the heat exchanger and the adsorbent, significantly increases the efficiency of adsorption and desorption in the bed. This allows reducing the size of the device without affecting the efficiency of the entire system. Therefore, to fill gas spaces in the vicinity of the heat exchanger, a material with higher thermal conductivity is required in which chemical and physical properties will not adversely affect the parameters of sorption phenomena occurring in the sorbent bed.

#### 1.2.4. Reducing the Thermal Capacity of the Bed

One of the ways to increase the cooling efficiency by increasing the COP is to reduce the heat capacity of the bed and, above all, the heat exchanger on which the adsorbent material is deposited. A group of scientists from Friedrich-Alexander-Universität in Erlangen-Nürnberg proposed using materials with reduced heat capacity for the heat exchanger [12]. The authors proposed the use of a polymer with increased thermal conductivity called poly(tetrafluoroethylene) coated with zeolite and studied the effect of aluminum admixture on its heat capacity, conduction coefficient, and water adsorption time in the bed covering this material. The obtained results turned out to be promising because the heat capacity of such material was reduced compared to pure aluminum by 30% with a mix containing 40% aluminum (which corresponded to the sample with the highest thermal conductivity tested).

The presented paper focuses on the selection of appropriate adhesives and binders that will increase the efficiency of heat exchange between the silica gel and the exchanger. Silica gels have low thermal conductivity (about 0.175 W/mK). One of the methods leading to the improvement of heat transfer properties is the use of a coated layer of silica gel mixed with a binder. The bonding of the boundary layers of the sorbent to the heat exchange surface through an adhesive improves the heat transfer conditions in the bed by significantly reducing the contact resistance at the gel border–heat exchanger boundary [13].

#### 1.2.5. The Use of Glues and Binders

**Figure 2:**
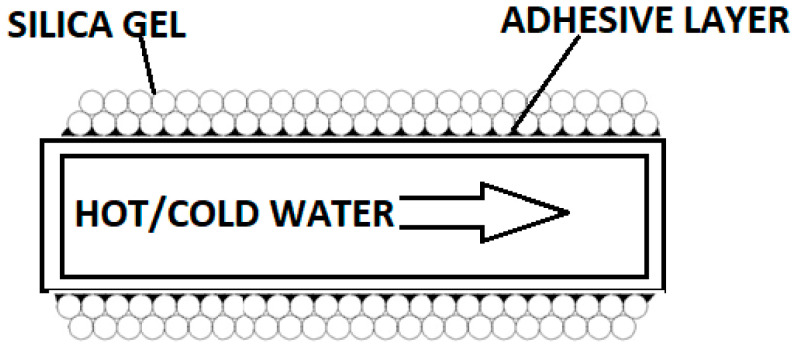
Cross-section of a heat exchanger with a silica gel coating (glued silica gel particles) [8].

Another way to increase the COP is to use a coated bed construction, where the adhesive layer allows reducing the porosity in the boundary layer of the heat exchanger [14,15]. In this configuration of the adsorption bed, optimal parameters, due to heat and mass transfer, depend on the valid selection of the sorbent–adhesive pair. The selection of adsorbent–glue–adsorbate components for the construction of the layered bed must be determined based on many tests and operating parameters. The analysis of thermal properties allows the correct selection of heat and mass transfer elements concerning improving the thermal conductivity of the bed. Figure 2 shows the cross-section of a heat exchanger with a silica gel coating.

The performed analysis aimed to determine the amount of moisture adsorbed from the air by a representative sample of a coated bed; an aluminum heat exchanger covered with glue and an adsorbent layer. Various types of glue were used for the tests. The addition of fine silica gel should enhance bed packing, allow a reduction in the overall size of the cooling unit, and decrease glue surface stress, leading to better adhesive distribution on the heat exchanger surface.

Based on the analysis of the glue working conditions, the desired properties can be determined. One of the criteria is to maintain mechanical strength within bed operating temperatures despite periodic changes in relative humidity. High values of adhesive forces between the sorbent and the heat exchanger surface and the stability of parameters during many operation cycles are also of great importance.

The next criterion is the heat transfer coefficient of the glue. Taking this parameter into account allows to conclude that epoxy resins or vinyl polymers, including e.g., PVA or Wikol^®^, can be potentially used as adhesives in adsorption chiller beds. These adhesives are characterized by high bond strength in environments with high humidity. The benefitial effect of polyvinylpyrrolidone coating on thermal conductivity was described in [16]. The value of the thermal conductivity of silica gels deposited on PVP—poly (vinylpyrrolidone) adhesive was 0.28 W/mK [8]. This value is 60% higher than that obtained using sorbent without the binder. Epoxy resins have a thermal conductivity much higher than 2 W/mK, good water resistance, and high mechanical strength. Manufacturers also guarantee their stability at temperatures above 400 °C. When it comes to thermal conductivity, groups of adhesives based on epoxy resins modified with the addition of powdered metals are also distinguished [17,18].

Modified resins are dedicated to systems where it is crucial to ensure adequate heat conduction and removal of air bridges. The value of thermal conductivity for this group of resins is 5 W/mK. These materials are still not used as adhesives in adsorption beds due to the lack of appropriate tests and adaptation of mixtures to working conditions. The study determined the amount of adsorbed moisture from the air and the structure of the sample surface with particular emphasis on the adsorbent surface.

Many recent studies are dedicated to the maximization of adsorption chiller cooling efficiency by an adsorbent modification to improve its thermal conductivity. Some research determines the metal additives effect [18], other deals with composites [18,19,20,21,22] and the use of coatings [23,24,25,26]. In this study, a comparative study of adhesives—commercially available glues was performed. To determine the effect of silica gel-glue on the adsorption processes, a number of experimental studies involving the use of coated beds were performed. The study determines the influence of adhesive and preparation method on modified sorbent sorption properties.

## 2. Materials and Methods

### 2.1. Materials

The adsorbent used was silica gel with a grain size of 1000 μm and <200 μm added to the adhesive.

The adhesives used for testing are:

Glue 1: Poly(vinyl alcohol) at a concentration of 5%;
Glue 2: Hydroxyethylcellulose at a concentration of 1.25%;
Glue 3: 2-Hydroxyethylcellulose at a concentration of 5%.

Dry silica gel can be mixed manually or using laboratory stirrer with adhesives and then dried at room temperature for about 20 h or at temperatures of about 75 °C for 15 min.

Poly(vinyl alcohol) is usually prepared as a water solution which should be prepared in an anticorrosive vessel. First, poly(vinyl alcohol) was mixed with cold water and then heat up to 90–95 °C in a water bath or using water vapor. The solution should be stirred (manually or with laboratory stirrer) throughout the whole process of heating up and cooling down, at a maximum of 4 h in total. Hydroxyethylcellulose glues were mixed with water at 25 °C and the temperature was kept still during the whole process by means of a laboratory heater. The maximum stirring time is 2 h.

### 2.2. Samples Preparation

The tested samples were put on round aluminum plates imitating the heat exchanger element placed in the bed. The plates with a diameter of about 9 cm had to be weighed and cleaned with alcohol in order to degrease and apply the adhesive layer as accurately as possible. Grease and dirt on the sample reduce the contact surface of the heat exchanger with the adhesive or adsorbent and thermally isolate the heat exchanger from other elements of the sample. The adhesive in the desired amount was manually applied together with the adsorbent and then the mixture was immediately spread over the entire surface of the metal tray. In order to increase the repeatability of the tests, the amount of glue and adsorbent materials was similar and the adsorbent was distributed uniformly to obtain a single-layer coating. The study aims to compare different samples and select the best components. Therefore, the samples differed in the adhesive as well as the granularity of the adsorbent and the method of drying the sample.

In this study, 12 samples were prepared for drying tests (see Table 1). The samples were prepared using:

- Three different types of glues;
- Two drying methods;
- Two different granulations of adsorbent.

For better visualization of the silica gel adhesive, a dye (red food coloring and red chalk) was added to the adhesive samples. Then, the tested samples were examined under a microscope to analyze the structure and nature of the adhesive coating of adsorbent particles. Samples with a dyed adhesive were observed under an optical microscope with a 150× magnification.

### 2.3. Methods

The samples were weighed several times during the moisture content test. First, the aluminum weighing pan was weighed, then the amount of glue and adsorbent used to create the sample was determined. Before measuring out the adhesive and adsorbent, both were taken from closed containers that had the shortest possible contact with the room air. If the adsorbent comes into contact with air for too long, it absorbs moisture and increases the mass and the adhesive dries, reducing it. Too much contact of the reagents with air would change their proportions and the concentration of the applied adhesive.

After weighing the reagents and mixing them, drying started to remove moisture from the adhesive and fix the adsorbent on the surface of the pan. Half of the samples were dried at room pressure and temperature, and a half was calcined at about 75 °C. Then, the samples were placed in a humid environment, in a desiccator for about 10 min, in stable conditions: 19 °C and 30–32% humidity. The samples were individually transported to a programmed weighing dryer, which examined the change in sample mass during drying. The drying temperature was 85 °C and the experiment end was programmed when the sample mass did not change for 25 s, assuming the samples are sufficiently dry. Sample weight measurements were recorded every 10 s until drying was completed. Such measurement allowed checking not only the moisture content but also the desorption dynamics.

## 3. Results and Discussion

In order to determine the effect of silica gel–glue on the adsorption processes, several experimental studies involving the use of coated beds were conducted. The mass of the adsorbent was selected in the amount necessary to cover the largest possible surface of the sample pan but limited at the same time by the risk of multiple layers formation. The amount of glue used was the minimum amount necessary to cover the entire adsorbent layer of the metal tray, the exact mass of the glue in each test is shown in Table 1. Most of the samples were successfully created. The noticeable large cracks in the adsorbent layer (sample material not in contact with the weighing tray), making the samples unsuitable for use in the bed, were observed only in the case of two samples: 8 and 11. The rest, however, does not show any signs of reduced strength and is in contact with the weighing pan; also, they do not crumble and do not break down (Table 2).

### 3.1. Sorption Properties of Modified Silica Gel (SG) Samples

For comparative purposes, several tests and calculations were carried out to determine comparable values for samples and to determine their characteristics. An essential comparative value is the amount of adsorbed water. At the beginning, the water adsorption capacity of produced modified silica gel (SG) samples and raw silica gel (SG) were determined. Based on measurements of the initial (dry) and final (before drying, at saturation conditions at 19 °C and 31% humidity) mass on parameter α, the moisture-to-adsorbent mass ratio was calculated according to Equation (1):

(1) $$\alpha =\frac{m_{w}}{m_{SG}}$$

where *m_w_* is mass of moisture adsorbed by given sample, g; and *m_SG_* is mass of silica gel in given sample, g.

**Figure 3:**
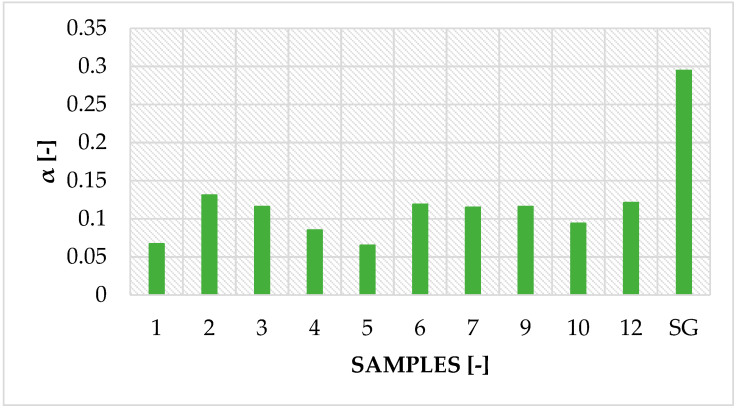
Moisture adsorption capacity of modified silica gel (1–12) and unmodified silica gel (SG) samples.

The result of the calculations are shown in Figure 3. Additional measurements were taken for the unmodified silica gel.

The adhesive reduces the active surface of SG crystals, which can be seen by comparing glued samples to samples with SG alone, for which the average moisture content to SG weight was about 0.295 (-). This is expected because the sample weight is reduced by the weight of the adhesive, which has a relatively small active surface.

The adhesive cannot negatively affect the silica gel sorption properties, as COP depends on sorbent adsorption capacity [8]. The properly selected adhesive affects sorbent sorption properties to a minor extent [26]. Additionally, a long-term test was done, and after placing the samples in a humid atmosphere for 24 h, only Samples 1–4 showed a moisture-to-adsorbent mass ratio similar to unmodified silica gel (SG). They were defined as the most promising adhesive–adsorbent pairs.

**Figure 4:**
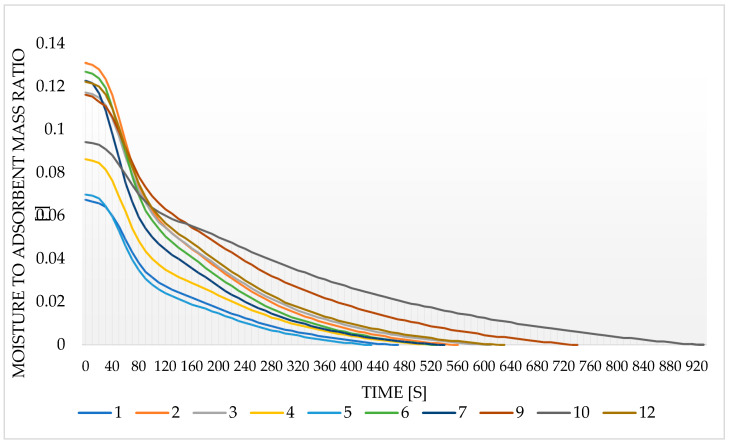
The change in the ratio of the mass of moisture contained in the sample to the mass of the adsorbent during drying.

The results of the desorption kinetics determined based on continuous measurements in the weighing dryer are presented in Figure 4. The curves correspond to sample numbers in Table 1.

It can be seen that the largest amount of moisture in a short adsorption time (10 min) in relation to the SG used can be observed in Samples 2, 12, and 6, where the amount of moisture mass per SG mass is around 0.12–0.13 (-). It should be noted that all these samples were surface dried. For Samples 5, 1, and 4, the discussed coefficient was in the range of 0.065–0.085 (-). Samples 1 and 5 are calcined samples with no addition of fine SG to the glue, which may indicate techniques producing lower quality samples. An additional test for the pure SG sample was performed, and as expected, the highest value of the moisture-to-adsorbent mass ratio was noted as equal to 0.29 (-). The sample was completely dry after 796 s but the curve was not added in Figure 4 so as to not decrease its readability.

Modified sorbents with the best characteristics are characterized by a significant initial moisture weight reduction, followed by a relatively dynamic reduction of this parameter. In the case of the sample with the highest initial moisture weight reduction, the following relatively dynamic reduction of this parameter suggests that the technique for the preparation of Sample 2 is more useful than the technique used for Sample 12, which, despite a relatively high initial coefficient, has a slower desorption rate. Due to the adopted working time model, the dynamics may be more important than the initial amount of adsorbed moisture. While the initial moisture content is higher in Sample 10 than in Sample 5, the desorption process is more dynamic in Sample 5 and therefore it is possible to shorten the adsorption and desorption cycle times and thus increase the amount of transferred energy over a longer period, i.e., to perform more cycles at the same time.

It is clearly visible that samples glued with Adhesive 3 (Samples 1 to 4) are heavily coated with the glue, which can hinder moisture adsorption on SG particles with a small contact area with the moisture. In addition, fairly good adhesion of the particles together can justify a relatively high desorption dynamics because the particles glued on with a heat-conducting adhesive heat up faster, while their adsorption is more dynamic due to the smaller contact surface between SG and water vapor. Adhesive 3 also seems to have high surface tensions, which results in good adhesion and tight gluing of the SG particles, but may suggest a lower extent of penetration of the pores of the SG particles, which would reduce its active surface. The latter claim seems to be confirmed by the research results, because samples glued with this adhesive seem to have the highest water vapor adsorbing capacity. Adhesive 1, which was used to prepare Samples 5, 6, and 7, covers SG similarly to Adhesive 3. Samples prepared with the use of the mentioned adhesive have a relatively high desorption dynamics, which may be responsible for the high density of the adhesive, and moisture storage capacity, which suggests a low level of SG pores filling with the adhesive agent. On the other hand, the thinner adhesive layer seems to have less effect on the dynamics of spontaneous adsorption of moisture from the environment, as the measurements of the moisture after 10 min and 24 h were similar.

### 3.2. Observation of Prepared Samples under the Optical Microscope

**Figure 5:**
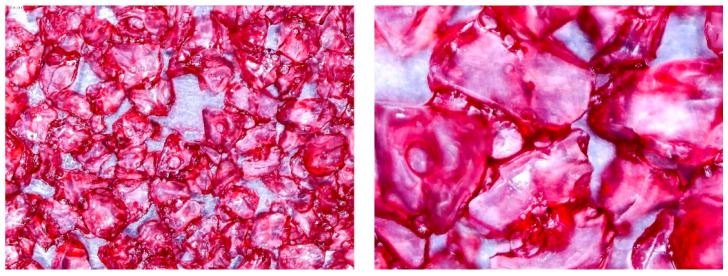
(**Left**) Silica gel sample mixed with Glue 1 (poly(vinyl alcohol)) and (**Right**) red food coloring.

**Figure 6:**
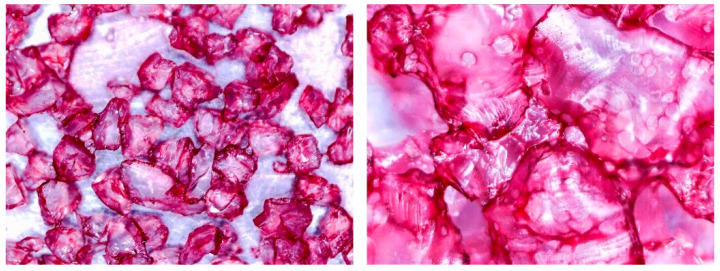
(**Left**) Silica gel sample mixed with Glue 2 (hydroxyethyl cellulose)) and (**Right**) red food coloring.

**Figure 7:**
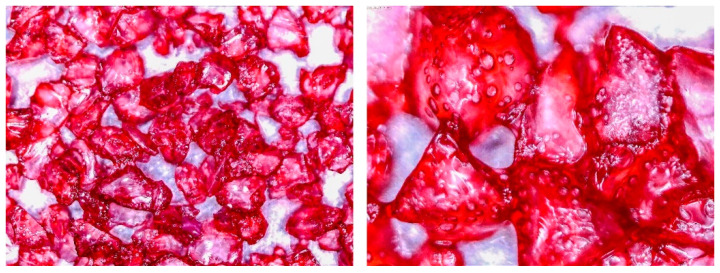
(**Left**) Silica gel sample mixed with Glue 3 (2-hydroxyethyl cellulose) and (**Right**) red food coloring.

**Figure 8:**
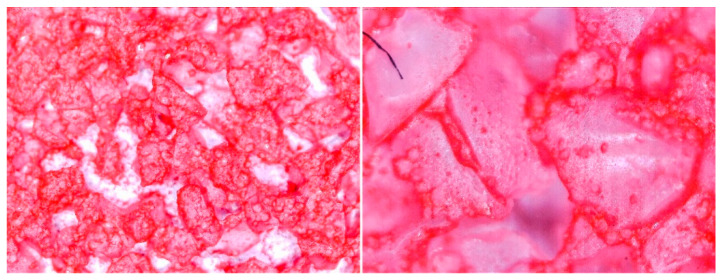
(**Left**) Silica gel sample mixed with Glue 1 (poly(vinyl alcohol)) and (**Right**) red chalk.

**Figure 9:**
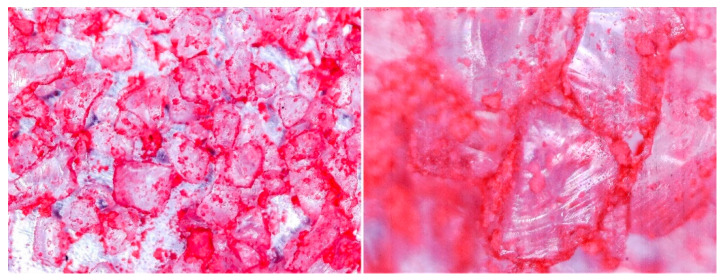
(**Left**) Silica gel sample mixed with Glue 2 (hydroxyethyl cellulose)) and (**Right**) red chalk.

**Figure 10:**
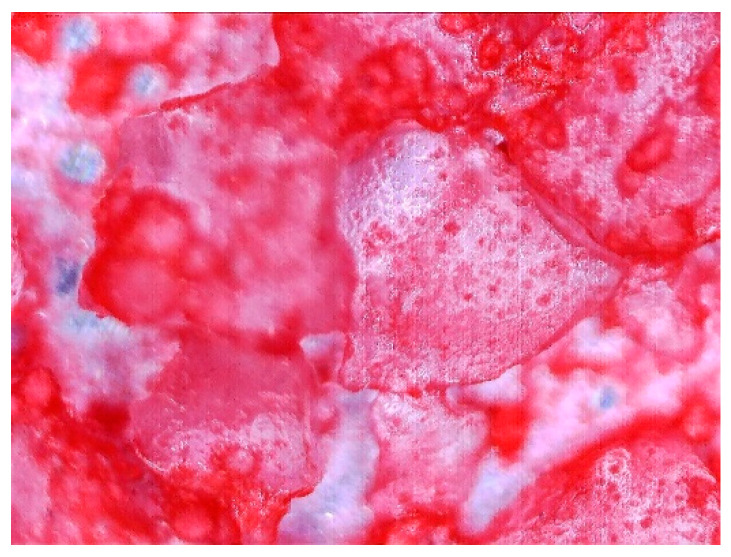
Silica gel sample mixed with Glue 3 (2-hydroxyethyl cellulose) and red chalk.

To determine the quality of adhesive coating on silica gel and the given glue adhesion on the surface of the adsorbent, the samples mixed with red food coloring and red chalk were observed under an optical microscope (Figure 5, Figure 6, Figure 7, Figure 8, Figure 9 and Figure 10).

Pictures taken with an optical microscope show that samples glued with Adhesive 3 (Figure 7 and Figure 10) are heavily coated with glue, it can limit moisture adsorption on SG particles that have a small contact surface with adsorbate (in this case humid air). Besides, quite good coverage and gluing of the particles together may justify a relatively high dynamics of desorption, as the particles are in close contact with the heat exchanging surface, but also a lower dynamic of spontaneous adsorption due to smaller contact surface of SG with water vapor. Adhesive 3 also seems to have high surface stress, it is rather well connected and quite tightly surrounds SG particles, but this may suggest that in a smaller alloy, it soaks into the pores of the SG molecule, which would reduce its reactive surface. It was also confirmed by the drying test results, as the samples glued with this glue seem to be the most capacious when it comes to the possibility of adsorbing water vapor.

Adhesive 2 (Figure 6 and Figure 9) seems to look different under the microscope than Adhesive 3. It seems less airtight when it comes to the SG coating. This would increase the dynamics of free adsorption. At the same time, the rare distribution of the adhesive results in lower desorption dynamics and the samples drying time was the longest. It is also possible that Adhesive 3 penetrates into the pores of SG crystals to a significant extent, reducing the active surface, and these samples had some of the lowest initial moisture contents.

Adhesive 1 (Figure 5 and Figure 8) seems to cover SG in a similar way to Adhesive 3 based on microscope images, but appears to have a thinner layer. Samples made with it also have a fairly high desorption dynamics, which can cause a high density of glue, and a high degree of moisture storage, which suggests a low level of clogging SG pores by the glue. On the other hand, the thinner adhesive layer seems to have less impact on the dynamics of spontaneous adsorption of moisture from the environment.

### 3.3. The Average Desorption Power

The average desorption power determines the amount of energy that the moisture contained in the sample is able to adsorb and refers it to the time of the desorption process. This parameter includes not only the amount of transferred energy but also, partially, the dynamics of the desorption process. The average desorption power in this study was calculated as follows:

(2) $$P=\frac{E}{t_{d}}$$

where *P* is average desorption power, W; *E* is energy necessary for moisture evaporation, kJ; and *t_d_* is drying time, s.

Knowing the difference between enthalpies of two following measuring points in apparatus working conditions it is possible to calculate the energy needed to transfer between these states, it means the amount of energy necessary to evaporate or heat up the moisture of a known mass in the samples, calculated as:

(3) $$E=dh\cdot m_{w}$$

where *dh* is a difference in enthalpy of two following measuring points, kJ.

**Figure 11:**
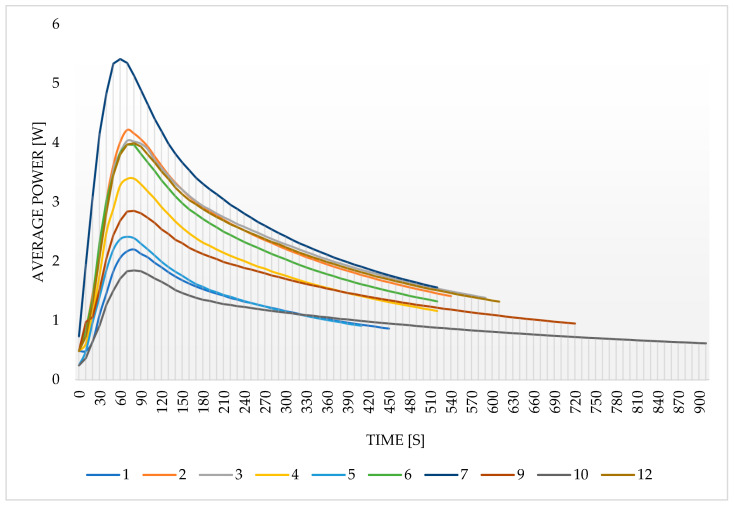
The change in average power over time.

Figure 11 shows the change in average power over time. It means that each subsequent point of the chart was calculated to determine the average desorption power the sample would have if the measuring would finish at this particular moment.

The curve shows that the drying of Sample 7 takes place significantly more dynamically than the drying of other samples, even though this sample did not have the highest adsorbed moisture among the tested samples. Besides, Sample 10 seems to perform the least dynamic desorption despite a relatively high moisture content at the beginning. The next samples with low desorption dynamics are Samples 1 and 5, where, according to the graph, showed the dynamic change of the fraction of residual moisture. Their low average power values result from the relatively small amount of moisture that they adsorbed before the measurement; therefore, after solving the problem of low moisture of the sample, it can be expected that their desorption dynamics should be significantly improved. The capacity of Bed 1 is high, while the low moisture content is due to the low dynamics of spontaneous adsorption; the sample, after a longer adsorption time, should have relatively better desorption dynamics.

In a real unit, the moment of desorption completion and bed adsorption start is adjusted, which may change the average desorption power for samples, especially since the final drying of the samples takes place slowly and seems to be not very profitable. Naturally, more accurate drying of the samples can translate into a more efficient and dynamic adsorption process, but the adjustment of the cycle time should take into account the desorption time required for the effective course of the process. Therefore, the graph of the average power change during the desorption process can help in choosing the cycle time scheme.

## 4. Conclusions

A total number of 12 samples was prepared, two of them were rejected due to their fragility and lack of adherence to the metal plate resembling a heat exchanger. The samples were glued with three different adhesives. Then, the samples were placed in a desiccator for 10 min to imitate uniform conditions for moisture adsorption. Then, samples were dried in a moisture analyzer, continually recording sample weight changes.

The results suggest that the choice of glue is essential for the characteristics of the bed. Based on the results, it can be determined, for example, how dynamically free adsorption takes place, how much moisture the sample can adsorb, or what the desorption dynamics are. Samples 1–4, made with the use of Adhesive 3 (2-hydroxyethyl cellulose), show high adsorption capacity, low dynamic adsorption, and medium dynamic desorption. Samples 5–7, containing Adhesive 1 (poly(vinyl alcohol)), adsorb slightly less moisture, but their free adsorption and desorption seem to be more dynamic. Samples 9–12, containing Adhesive 2 (hydroxyethyl-cellulose), seem to show lower moisture capacity, relatively dynamic adsorption, and lower dynamic desorption. Besides, the desorption of Samples 1, 5, and 7 seem to be the most dynamic, Samples 3, 2 and 4 show the most significant adsorption capacity, while Samples 10, 12, 6, and 7 show the most dynamic free (non-forced) adsorption. The adhesive used for the production of the bed is of great importance; however, any changes in the concentration of the adhesive, sampling technique, the amount of adhesive, or SG grain size in the sample will affect the parameters and suitability of the sample. The microstructural assessment of adsorption phenomena was carried out by means of the optical microscope and it is the first stage to indicate a possible mechanism of adsorption. The presented results are the preliminary studies performed to select the most promising glue–silica gel pairs and ratios that will be further tested using a vacuum kinetics analyzer.

## Figures and Tables

**Table 1:** Sample list with adsorbent and adhesive masses and observations after sample preparation on metal trays.

No.	Glue Mass (g)	SG Mass (g)	Glue Number and Concentration	SG Sample and Drying Procedure	Observations
1	4.317	2.417	3/5%	SG 1000, drying 75 °C	Multilayered on the edges
2	4.011	2.428	3/5%	SG 1000, drying 20 °C	Big noncovered spaces
3	4.44	2.930	3/5%	SG 1000 + SG < 200, drying 75 °C	Covered tight
4	4.41	2.952	3/5%	SG 1000 + SG < 200, drying 20 °C	Irregular thickens of the layer
5	4.083	2.401	2/1.25%	SG 1000, drying 75 °C	Big noncovered spaces
6	3.895	2.407	2/1.25%	SG 1000, drying 20 °C	Multilayered on the edges
7	4.425	2.927	2/1.25%	SG 1000 + SG < 200, drying 75 °C	Wholes from water evaporation
8	4.435	2.444	2/1.25%	SG 1000 + SG < 200, drying 20 °C	Porous, many cracks and weak adherence
9	3.57	2.462	1/5%	SG 1000, drying 75 °C	Locally high density
10	3.573	2.766	1/5%	SG 1000, drying 20 °C	Irregular density
11	3.55	2.417	1/5%	SG 1000 + SG < 200, drying 75 °C	Porous, many cracks and weak adherence
12	3.57	2.428	1/5%	SG 1000 + SG < 200, drying 20 °C	Irregular thickens of the layer

**Table 2:** Samples prepared for adsorption–desorption tests.

No.	Silica Gel Mixed with Adhesive on A Metal Tray Resembling A Heat Exchanger	No.	Silica Gel Mixed with Adhesive on A Metal Tray Resembling A Heat Exchanger
1	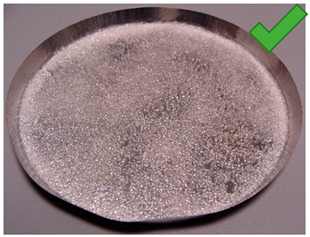	7	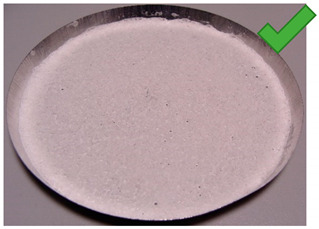
2	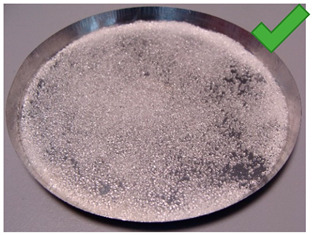	8	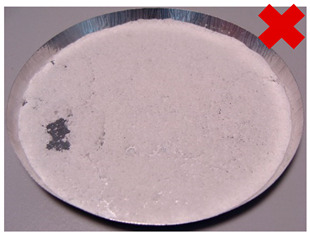
3	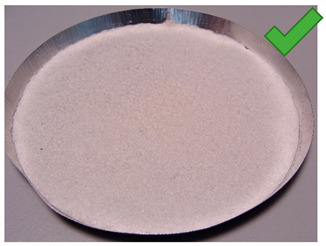	9	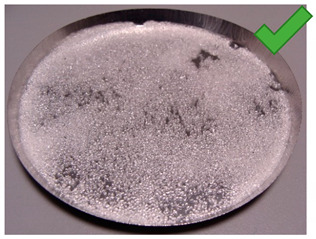
4	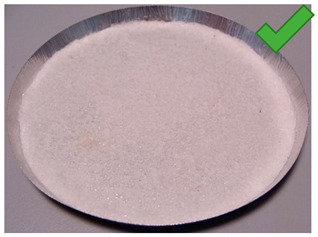	10	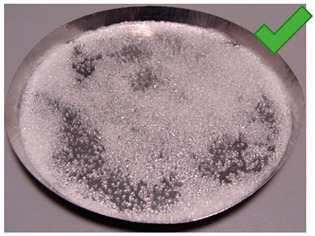
5	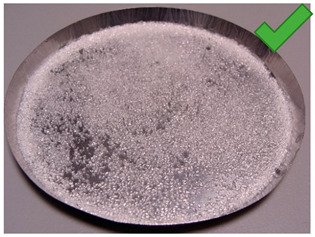	11	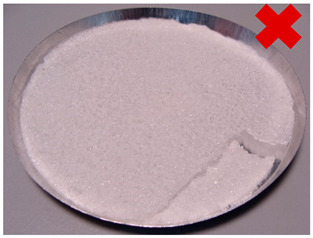
6	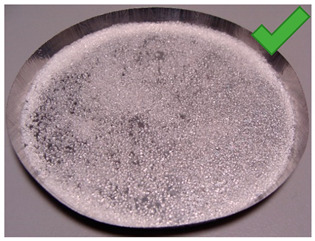	12